# An End-To-End LwM2M-Based Communication Architecture for Multimodal NB-IoT/BLE Devices

**DOI:** 10.3390/s20082239

**Published:** 2020-04-15

**Authors:** Subho Shankar Basu, Jetmir Haxhibeqiri, Mathias Baert, Bart Moons, Abdulkadir Karaagac, Pieter Crombez, Pieterjan Camerlynck, Jeroen Hoebeke

**Affiliations:** 1 IDLab, Ghent University—imec, 9000 Ghent, Belgium; jetmir.haxhibeqiri@ugent.be (J.H.); mathias.baert@ugent.be (M.B.); bamoons.moons@ugent.be (B.M.); abdulkadir.karaagac@ugent.be (A.K.); jeroen.hoebeke@ugent.be (J.H.); 2 Televic Healthcare, 8870 Izegem, Belgium; p.crombez@televic.com (P.C.); p.camerlynck@televic.com (P.C.)

**Keywords:** multi-modal architecture, handover, narrowband internet of things (NB-IoT), bluetooth low energy (BLE), light-weight machine to machine (LwM2M)

## Abstract

The wireless Internet of Things (IoT) landscape is quite diverse. For instance, Low-Power Wide-Area Network (LPWAN) technologies offer low data rate communication over long distance, whereas Wireless Personal Area Network (WPAN) technologies can reach higher data rates, but with a reduced range. For simple IoT applications, communication requirements can be fulfilled by a single technology. However, the requirements of more demanding IoT use cases can vary over time and with the type of data being exchanged. This is pushing the design towards multimodal approaches, where different wireless IoT technologies are combined and the most appropriate one is used as per the need. This paper considers the combination of Narrow Band IoT (NB-IoT) and Bluetooth Low Energy (BLE) as communication options for an IoT device that is running a Lightweight Machine to Machine/Constrained Application Protocol (LwM2M/CoAP) protocol stack. It analyses the challenges incurred by different protocol stack options, such as different transfer modes (IP versus non-IP), the use of Static Context Header Compression (SCHC) techniques, and Datagram Transport Layer Security (DTLS) security modes, and discusses the impact of handover between both communication technologies. A suitable end-to-end architecture for the targeted multimodal communication is presented. Using a prototype implementation of this architecture, an in-depth assessment of handover and its resulting latency is performed.

## 1. Introduction

By bridging our physical and virtual worlds and paving the way for interactions between humans and machines, IoT is finding uses in an increased number of applications. Devices using IoT are expected to be in the billions and the ability of wireless communication is an indigenous part of each of them. Each wireless communication technology has its own characteristics in terms of range, throughput, latency, reliability, downlink availability, security, energy consumption, device and deployment cost, etc. and it is not possible to get the best of all when picking a single technology. Also, different IoT applications have different requirements regarding these parameters. Thus, it is very important to choose the wireless communication technology as per the application demands. Many present day IoT applications are simple and stand-alone, having limited and static requirements. Here, a single communication technology suffices to their needs. However, in the near future with the increasing pace of IoT deployments, applications tend to become more dynamic where the requirements can vary over time and with the type of data being exchanged. This leads to the need for multimodal IoT solutions, where different wireless communication technologies are combined to satisfy the more demanding communication requirements and devices can switch from one technology to the other, also called a vertical handover. In this paper, we explore the combination of LPWAN and WPAN technologies, where LPWANs have the ability to communicate over very long distances at low data rates, whereas WPANs have higher data rates but are limited to shorter distances. More specifically, this paper considers the combination of NB-IoT, an LPWAN technology that is operating in licensed spectrum, and Bluetooth Low Energy (BLE), a widespread WPAN technology for short range communication. On top, from a protocol stack perspective, we assume the use of Lightweight Machine to Machine (LwM2M) [1] over Constrained Application Protocol (CoAP) [2] as common higher layers, in order to avoid having different application logic on a single device as well as over the various end-to-end communication paths. Heterogeneous devices from different vendors can still have their own software stack underneath, but by using a common higher level application technology such as LwM2M, an easy end-to-end communication architecture is established irrespective of the network and device heterogeneity. Taking this as a starting point, this paper analyses the challenges incurred by different protocol stack options and discusses the impact of handover between both communication technologies. From a protocol stack point of view, Internet Protocol (IP) has helped on a lot of occasions for pervasive communication over the Internet and has been the choice of network protocol for many communication technologies. However, due to its header overheads (~20–40 bytes), small-sized data applications (~few bytes) find it inefficient. Furthermore, some wireless technologies (e.g., Sigfox) allow only a few bytes (~8–12 bytes) of payload making the delivery of IP datagrams impossible and leaving Non-IP Data Delivery (NIDD) as the only choice. Both NB-IoT and BLE support IP and NIDD and different combinations will have an impact on the end-to-end communication architecture. Furthermore, multimodal support increases the complexity of the device and the network. The device needs to be able to switch between the technologies, resulting in signaling and management communication overheads and leading to extra latencies and energy consumption. On top, due to the heterogeneous nature of the devices, networks and applications, an inter-operable architecture needs to be in place for a smooth and transparent functioning.

To address these challenges, a multimodal architecture has been designed with support for both NB-IoT and BLE. Moreover, support has been added for both IP and non-IP data delivery over these technologies. Static Context Header Compression (SCHC) [3] has also been embedded as a possibility to send compressed IP packets over non-IP, eventually having end-to-end IP data delivery. To not ignore the security aspects, Datagram Transport Layer Security (DTLS) [4] has also been embedded in the architecture. A prototype of the architecture has been implemented and evaluated, focusing on the latency overhead due to the handover between the possible modes (Technology, IP/non-IP, SCHC compression, and DTLS) leading to applications to choose the most effective mode as per their needs.

As such, the contribution of the paper consists of the architectural design and implementation of a single stack solution for supporting multimodal technologies and of being able to switch between them in an intelligent way. To the best of our knowledge, this work is one of the first initiatives to form and validate a uniform protocol stack to handle multimodal technologies on the same device with dynamic switching among them.

The paper has been organized as follows. Section 2 gives some brief background information on the technologies used and the concept of multimodal communication, followed by highlighting some of the related works in this direction in Section 3. Section 4 introduces our designed multimodal architecture, as well as the realization of an end-to-end setup for validation and experimentation purposes. Next, an evaluation of the system is provided in Section 5, focusing on the impact of handovers on latencies. This is followed by a further analysis of the behavior in Section 6, thereby proposing some suggestions on how to use the system. Last, all findings are brought together in Section 7, illustrating the handover concept for an example application scenario, to finally conclude and give some directions for future work in Section 8.

## 2. Background and Problem Statement

This section gives a brief explanation of the two technologies used, the IoT protocols under consideration for the IoT device and a discussion on the challenges and problems faced with when considering handovers between both technologies by means of an example scenario.

### 2.1. Wireless Communication Technologies

#### 2.1.1. NB-IoT

**Figure 1:**
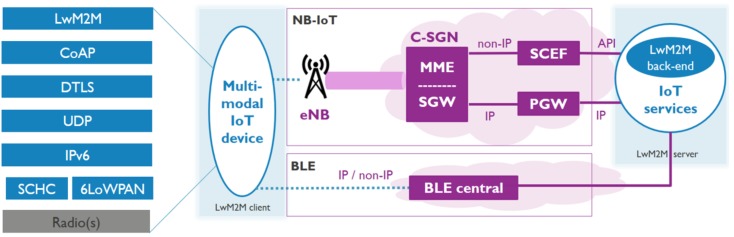
Envisioned multimodal communication architecture, leveraging on standardized IoT protocols and able to deal with the specific wireless communication peculiarities.

NB-IoT is an LPWAN technology from the 3GPP specification targeted towards low power and long range IoT applications [5]. It operates in the licensed spectrum using the LTE infrastructure in its simplest form of a single resource block. This results in cheap hardware and deployment costs, but still provides a decent service for a large number of IoT devices. Real performance evaluations of NB-IoT show that it can provide a throughput of at least 10 kbps for both uplink and downlink in good channel conditions and can penetrate deep indoor with no packet loss [6,7]. The power consumption of NB-IoT is also low (~70–220 mA for TX and ~46 mA for RX) and even more power can be saved by transitioning into Power Saving Mode (PSM) (~3 μA) as inferred from [8]. Moreover, when not in PSM, devices are always reachable for downlink communication favoring actuating devices and applications. NB-IoT supports both IP and non-IP data delivery methods as shown in Figure 1. Non-IP Data Delivery (NIDD) uses the Non-Access Stratum (NAS) to forward data over the control plane to Service Capabilities Exposure Function (SCEF) which interfaces with the services on a server. IP packets are forwarded and follow the standard end-to-end architecture between the client and the server. Consequently, NB-IoT serves as a promising technology for low-power long-range IoT applications like smart parking, smart metering, emergency health services, evacuation services, etc.

#### 2.1.2. BLE

BLE belongs to the family of WPAN technologies. It is a descendant of Bluetooth classic working in the unlicensed 2.4 GHz band, but it conserves even more energy by increasing the connection interval on the peripheral device. As it works at a higher frequency, it can achieve higher data rates (~1–2 Mbps) but at the expense of shorter range (~100 m). The power consumption of BLE is also quite low with a peak current of ~5 mA [9]. BLE has very low latencies compared to LPWAN technologies, thus being applicable for data intensive applications over a short distance. BLE has its own native application stack, but extensions have been defined to function over IP as well, using the 6LoWPAN adaptation layer. BLE functions as a peripheral and central pair and can support different modes, with the connected mode further being considered used in this paper. Multiple BLE peripherals can be connected to a BLE central leading to a flexible architecture and ease of deployment. Therefore, this technology is perfect for indoor use cases like smart speakers, home entertainment, firmware updates, etc. The authors of [10,11] perform measurement studies with BLE involving the achieved data rates and power consumption in different commercial BLE devices with different BLE versions and varying configuration parameters to provide an outline for designing an optimal IoT application.

### 2.2. IoT Protocol Stack

#### 2.2.1. LwM2M

LwM2M is a common device management and service enablement platform for IoT devices and systems. It provides APIs for bootstrapping, registration, data access, and eventing. It runs over CoAP and defines a client-server architecture where a LwM2M client (CoAP server) registers itself to the LwM2M server (CoAP client) as an endpoint as shown in Figure 1. The LwM2M client has a number of objects (both default and user-defined) and each object instance has resources that can be read, written, modified, executed, and observed by the LwM2M server by performing CoAP request to well-defined URIs. Being a common standard and running on top of the standardized application layer protocol for IoT (CoAP), it helps to bridge the gap between heterogeneous devices and helps in a smooth interoperable environment. The specification also provides robust security mechanisms like DTLS that can run over UDP. Overall, LwM2M acts as a robust and convincing management and data flow platform for IoT devices and applications.

#### 2.2.2. SCHC

IP serves as the basis of all communication over the Internet, but also imposes header overhead. Therefore, for very small quantities of IoT data (few bytes), IP (minimum 20 bytes header) does not turn out to be very efficient. However, to really integrate IoT devices in the Internet or in order to reuse the same stack across different radio technologies, IP is still the preferred choice. This is where SCHC comes into play. It is a compression and fragmentation technology that is applied over a CoAP, UDP, and IP packets. SCHC maintains some synchronized static rules on the sender and receiver and the rules contain various header information. Each packet is compared against the rules and if a rule matches the packet’s header, the header is replaced by the rule ID thereby reducing the header size. Rules can also be partially matched where the matching part is ignored and only the non-matching part is included in the header. Best case, this makes it possible to reduce the header size from 32 bytes (CoAP + UDP + IPv4) or 52 bytes (CoAP + UDP + IPv6) down to 2 bytes (16 bits rule ID). The receiver retrieves the rule ID and uses it to reconstruct the header, thereby imitating virtual IP communication with reduced packet overhead between the SCHC enabled sender and receiver. This is very efficient for IoT use cases as most of the header information remains the same for these applications. At the same time, it offers a possibility to communicate over non-IP technologies as well, while maintaining end-to-end IP principles.

#### 2.2.3. DTLS

IoT use cases cannot ignore security, but as these devices have limitations on power consumption and computation capabilities, efficient security techniques need to be applied on them. Transport Layer Security (TLS/SSL) on top of TCP has been a long standing success for security over the Internet. However, as most IoT use cases run over UDP for power constraints, they rely on DTLS. DTLS authenticates the communicating entities and establishes the key for the secure encryption. DTLS can be deployed in three different credential types namely Pre-Shared Key (PSK), Raw Public Key (RPK), and the certificate mode [12]. In this paper, the effects of PSK and certificate modes have been studied to see the impact on handovers. In PSK mode, a pre-shared key is shared in advance between the sender and the receiver, and the master key is derived from it, therefore involving lower complexity, but reduced flexibility at times of a key compromise and generation of fresh keys. The certificate mode on the other hand exchanges certificates and establishes the master key by using asymmetric encryption and is thus more flexible towards key compromise, but is more computation and power hungry.

### 2.3. Multimodal Communication

Our targeted multimodal communication setting is depicted in Figure 1. It consists of the communication network in the center, the end-device at the left, and the back-end server at the right. The network, in our case NB-IoT or BLE, consists of the central communication backbone for data exchange between the device and the server. For NB-IoT, data exchange can happen both over IP and non-IP infrastructures between the device, eNB, CIoT Serving Gateway Node (C-SGN), and the back-end. For IP-based data exchange, the eNB forwards the packets to the Serving Gateway (SGW), which in turn forwards them to the PDN Gateway (PGW). The PGW then forwards the egress packets to the internet destined for the back-end. For non-IP-based communication, the eNB forwards the data to the Mobility and Management Entity (MME), from where it is forwarded to the Service Capability Exposure Function (SCEF). The SCEF then forwards the data to the Application Server via an API. Downlink data transfers happen along the same corresponding lines in the opposite direction for both IP and non-IP. For BLE communication, the peripheral and the central maintains a connection for data exchange. For IP based data exchange, the peripheral sends an IP packet to the central from where it gets routed to the back-end via the internet. For non-IP data exchange the message is sent from the peripheral to the central, where it is encapsulated in an IP packet and gets routed to the back-end over the internet. Downlink communications happen in the same way in the opposite direction.

**Figure 2:**
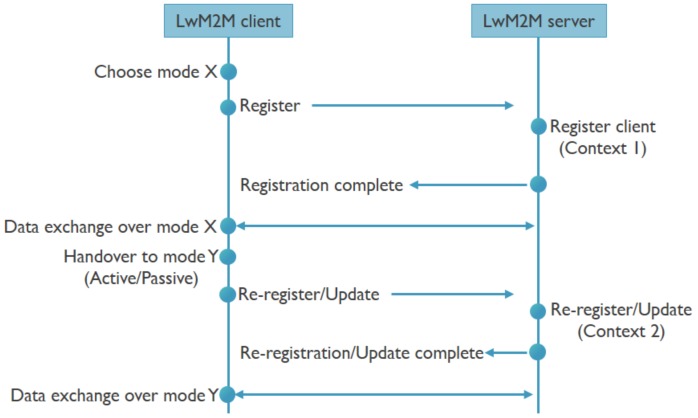
Handover in a Lightweight Machine to Machine (LwM2M) environment.

The multimodal IoT device has multiple communication technology radios, in our case NB-IoT and BLE, and an IoT protocol stack with LwM2M and CoAP running at the application layer. The device is able to communicate over the network with a LwM2M back-end, and is able to use either IP or non-IP based communication. This means that the device could pick any mode (Technology, IP/non-IP, SCHC, DTLS) to register itself with the LwM2M back-end and exchange data. Given the multimodal setting, the device can perform a handover to another mode. At this point in time, the LwM2M back-end should become aware of the new context (e.g., a different IP and port) in order to be able to continue to exchange data. To achieve this, the device can send either a re-registration or update message to the back-end. Once the new context is known, all further data exchanges now happen over the new mode. This is how the handover takes place smoothly and data exchanges are resumed. The process flow for a handover from a LwM2M perspective is shown in Figure 2. So far, the impact of such handovers on the required communication architecture, the involved signaling and resulting latencies has not yet been explored.

## 3. Related Work

In [13], Tervonen et al. states the importance of addressing heterogeneity in a WSN-based IoT application and to leverage on different communication technologies, co-operating among themselves using cognitive infocommunications. They also highlight the importance of energy constraints in IoT nodes and to address it by using energy harvesting techniques. Lopes et al. [14] proposes a multi-tier multimodal wireless sensor network for environmental monitoring and, with the help of simulations, shows that the model has much more benefits in terms of functionality, optimized resource usage, and energy consumption. Tunca et al. [15] proposes a multimodal system for a WSN framework in a home environment for assisted well-being, highlighting the advantages of multimodal systems in respect of the cumulative efforts that lead to a more powerful functionality which is otherwise not possible for unimodal systems. The authors of [16] describe with a real-life experimental validation the advantages of using connectionless BLE communication with the advertising mode thereby supporting more than 200 devices in the same region with a decent reliability and data rate for IoT applications. The authors of [11] study the differences in performance regarding throughput and energy consumption for different BLE versions and for varying configuration parameters. They show how the newer generations of BLE achieve better energy efficiency, data rates and coverage by means of real-life experiments. Aranda et al. [17] did some studies on the energy consumption of a WSN and compared Wake-up Radios with Duty Cycling Radios and their evaluations show that multimodal approaches lead to better optimizations in context awareness leading to lower energy consumption in these networks. All these works presented are either simulation based or only consider WPAN technologies, whereas this paper tries to accumulate the advantages of combining both WPAN and LPWAN technologies, along with an evaluation of a real implementation. Nonetheless, these works promote multimodality as a viable option for a wide number of use cases. Along this path, Famaey et al. [18] proposes a flexible architecture and implementation for heterogeneous wireless communication with the possibility of dynamic technology handovers. Their theoretical study shows the potential efficiency that can be gained in terms of flexibility and energy consumption by exploiting communication heterogeneity. Hoebeke et al. [19] discusses the need for a multimodal approach for IoT applications and comes up with a design of a virtual network operator (VNO) infrastructure with transparent access among heterogeneous networks, integrating the use of LwM2M and SCHC for uniform and efficient access to the end devices and network infrastructure. They only considered unlicensed LPWAN communication technologies. This paper leverages on the proposed VNO concept, with added support for the control and data plane and extends it with support for a licensed LPWAN technology (NB-IoT), as well as short range communication capabilities. Moreover, these works did not include an in-depth evaluation on the handover latencies and the interplay with LwM2M.

## 4. System Architecture and Realization

### 4.1. High-Level Architecture

**Figure 3:**
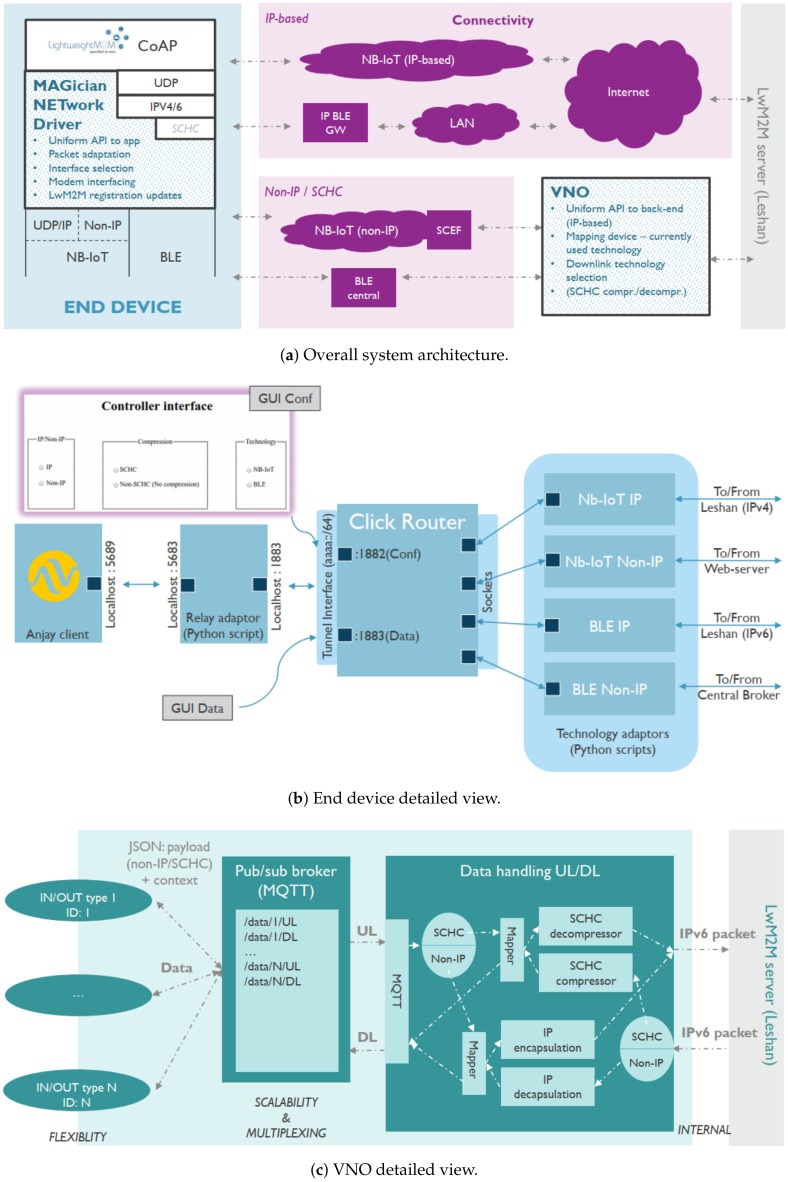
System architecture.

Figure 3a shows the complete high-level end-to-end system architecture, illustrating all possible communication modes that can be introduced in a multimodal NB-IoT/BLE setting. It consists of the end device, the network and the backend server. The end device is characterized by having two radio technologies, NB-IoT and BLE, which both support IP and non-IP data communication. Furthermore, the device runs a LwM2M client over CoAP at the application layer, providing a uniform API independent of the underlying technology. The LwM2M client deals with the registration, updates and data transfers. In combination with SCHC and DTLS this results in the following possible data transfer modes.

- Non-IP-based communication, where the LwM2M/CoAP payload is directly sent over the non-IP mode of BLE or NB-IoT, bypassing the other networking layers.
- Non-IP-based communication, where the complete IP packet is compressed using SCHC and transferred over the non-IP mode of BLE or NB-IoT.
- IP-based communication using either the IP mode of BLE or the IP mode of NB-IoT.

To handle the different possibilities and manage the underlying radios, the MAGician NETwork Driver (MAGNET) has been introduced. It offers a middleware layer that provides a standardized API towards the applications. After taking in the application layer data, it will perform the necessary packet adaptations to make the data ready for transmission over the selected interface. In order to perform proper interface selection, it will monitor the underlying technologies. Last but not least, if required, it will trigger the application layer to perform a LwM2M registration update.

Looking at the right side of the architecture, it is clear that in case IoT devices are allowed to use different communication technologies and modes, there should be an entity that will merge the traffic coming from both technologies/networks as well as decide which technology to use in downlink. For this we designed the Virtual Network Operator (VNO) concept, which handles all non-IP based interactions, including downlink interface selection, SCHC compression/decompression (if used), and which offers a uniform API towards an off-the-shelf LwM2M server with IP-only support.

The LwM2M server signifies the end-point of the north-bound interface where all the device management and data transfer can be controlled and accessed over a web based API. The IoT services on top are completely hidden from the underlying complexities of multimodal communication.

### 4.2. End Device Prototype

Figure 3b shows the realization of an end device prototype that offers the ability to choose between all possible modes, including a basic GUI for performing interface and mode selection. The prototype is made up of an off-the-shelf open source LwM2M client implementation, Anjay [20]. All traffic generated by Anjay is intercepted by a relay adapter and handed over to a packet processing instance, implemented in Click Router [21], to prepare the packet for transmission. It acts as the center of intelligence for both the control and data plane. It can select the technology (NB-IoT or BLE), mode (IP or Non-IP) and compression (SCHC enabled or disabled) options. Based on the options chosen, the packet is forwarded to the correct technology adaptor. There are four technology adaptors, one for NB-IoT IP, NB-IoT Non-IP, BLE IP, and BLE Non-IP. For NB-IoT IP, the Click Router instance hands over the LwM2M/CoAP payload to the NB-IoT module [8], which includes its own IP stack and will send the data over the operator network. For BLE IP, the entire IPv6 packet is handed over the IPv6-enabled BLE module [9], connected to a BLE gateway that will route the incoming data to the Internet. For non-IP communication, the packet processing instance will either perform SCHC compression or take the LwM2M/CoAP payload, depending on the chosen mode. For BLE non-IP, a default BLE connection is used towards a gateway, which is configured to interact with the VNO (see further). For NB-IoT non-IP, the data has to be passed to an NB-IoT module in NIDD mode which in the implementation has been emulated by the use of a webserver connected to the VNO.

### 4.3. VNO Prototype

As mentioned, all non-IP traffic coming from the multimodal end devices will end up at the VNO, before being handed over to the LwM2M server. The internals of the VNO are shown in Figure 3c. At the left side, a number of adapters are responsible for bidirectional interactions with the non-IP networks, i.e., the SCEF in case of NIDD for NB-IoT and the non-IP BLE gateways for BLE. These adapters are linked to a data handling unit, again implemented in Click Router, via a broker for scalability purposes. When non-IP data from IoT devices is entering this unit, a mapping is maintained between the device ID and the used communication technology and mode, in order to allow downlink communication. Next, in case SCHC is used, decompression is applied and the resulting IP packet is handed over to the LwM2M Leshan server [22]. In case only the LwM2M/CoAP data was transmitted, an IP packet is fabricated before handing it over to the LwM2M server. In both cases, the source IP addresses used are coming from a pool of IP addresses managed by the VNO. The other way around, downlink IP packets that need to go over a non-IP communication channel will be routed to the VNO, as the destination address is part of the pool of addresses managed by the VNO. Upon arrival in the VNO, packets coming from the LwM2M server are treated in a similar way, but now in reverse order.

## 5. Evaluation

**Figure 4:**
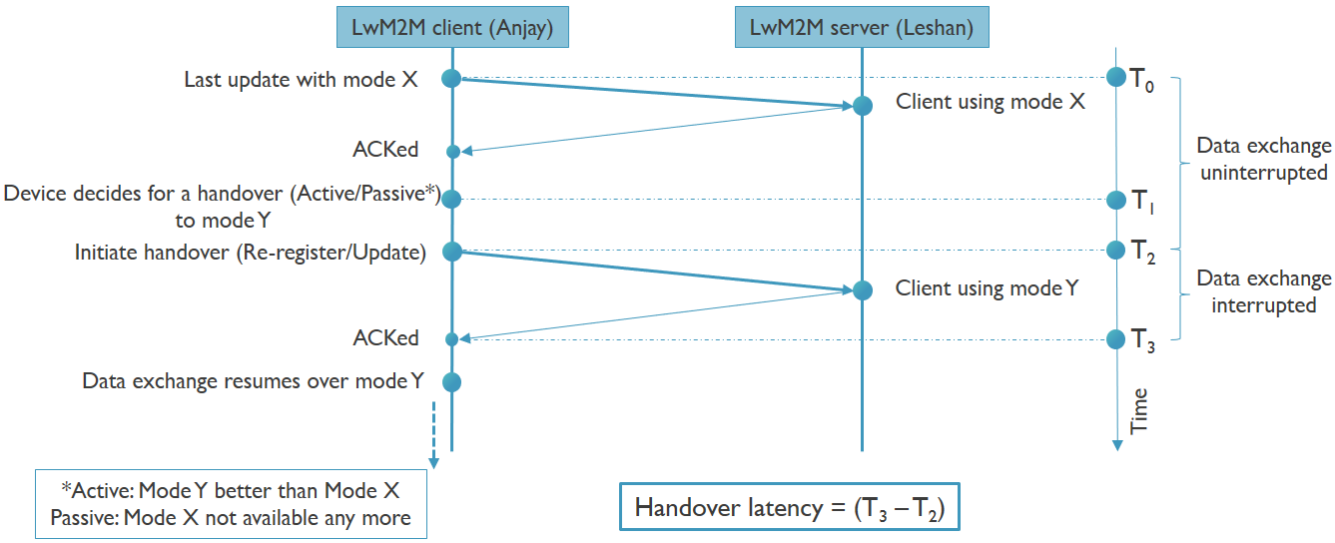
Handover diagram.

Using the presented setup, the impact of handovers between NB-IoT and BLE is evaluated. Apart from possible latencies introduced by activating the other radio or detecting the unavailability of connectivity over the current radio, another important contributing factor is the time it takes to re-establish the LwM2M context by performing a registration or update message from the LwM2M client to the LwM2M server as shown in Figure 4. This should be done for any handover between IP to non-IP and between different IP addresses, but also between different non-IP paths as the VNO needs to become aware of the path change. Therefore, this section explores in more depth this aspect. All modes of operation are considered, including IP, Non-IP, and SCHC for both NB-IoT and BLE. Moreover, the impact of using DTLS is taken into consideration. Further, a comparison has been done between the expected values and the actual values as obtained from the experiments, providing insights that are useful for predicting the latencies in different use cases. Table 1 shows the data sizes of different LwM2M messages in different directions for different modes.

### 5.1. NB-IoT Latency

The total time of a data transmission or reception over NB-IoT is given by

(1) $$NBIoT_{tx/rx}=t_{wu}+t_{sig}+t_{tx/rx}\cdot n_{rep}\cdot n_{TBs}+t_{cd}$$

where *wu* = Warm up, *sig* = Signalling, *n*${}_{TBs}$ = Number of Transport Blocks, *n*${}_{rep}$ = Number of repetitions, *cd* = Cool down, and *t* = Time. t${}_{tx/rx}$ denotes the time required for the transmission or reception of a single transport block. The number of transport blocks n${}_{TBs}$ can be calculated based on the data size and the MCS and TBS used during the data transfer. As the signal strength decreases, NB-IoT gradually increases the transmission power and reduces the MCS.

Based on this, the latencies of different LwM2M events were calculated as shown in Table 2. As can be noticed, the actual measured values are always greater than the expected ones (negative error rates) which is due to retransmissions, network scheduling, signaling latencies, processing delays, etc.

### 5.2. BLE Latency

The connected mode operation of BLE maintains a connection between the central and the peripheral. It has connection events at fixed connection intervals and data transfer can happen during these predetermined intervals. The connection interval is configurable and devices with infrequent data exchanges can be configured with larger connection intervals to save power. In the context of this paper, the connection interval has been configured to 7.5 ms. Moreover, in this implementation, a single packet of maximum 244 bytes of application payload can be transmitted within a connection interval, with BLE having its own overhead of 21 bytes in layer 2. Given these characteristics, the latency of BLE can be modeled as

(2) $$BLE_{tx}=I_{c}/2+⎣S/244⎦\cdot t_{ci}+\frac{\left(\left(S\;mod\;244\right)+H\right)\cdot 0^{0^{\left(S\;mod\;244\right)}}}{r_{PHY}}$$

where *I*${}_{c}$/2 is the average buffering time until the next connection interval, *t*${}_{ci}$ the connection interval time, *S* the application data size in bytes, *H* the header size in bytes, and *r*${}_{PHY}$ the physical layer data rate which in our setup is 1 Mbps. Further, we assume that 0${}^{0}$ = 1 and 0${}^{x}$ = 0, where x is a positive integer. Using this, the latencies of the different LwM2M events were calculated as shown in Table 3. It has been assumed that an uplink LwM2M request and its downlink LwM2M response are separated by 30 connection intervals which is due to the network delays, processing delays at the server and serial communication delays at the device side. The results show that the expected values are within 15 percent of the measured average values. Positive error rates indicate over-estimated values and negative error rates indicate underestimated values.

### 5.3. Re-Registration Latency

**Figure 5:**
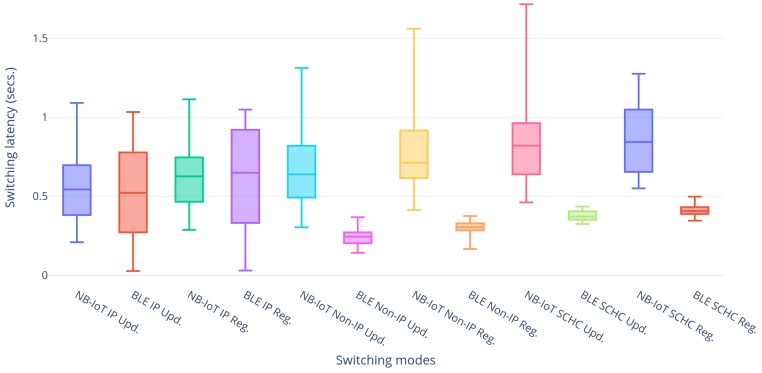
Handover latencies of Narrow Band IoT (NB-IoT) and Bluetooth Low Energy (BLE).

**Figure 6:**
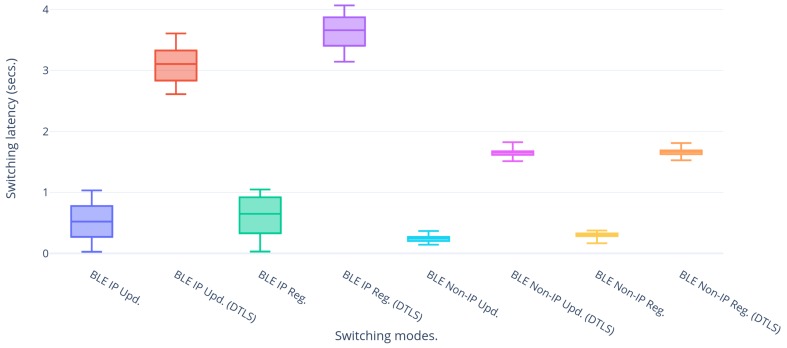
Handover latencies of BLE with(out) Datagram Transport Layer Security (DTLS).

**Figure 7:**
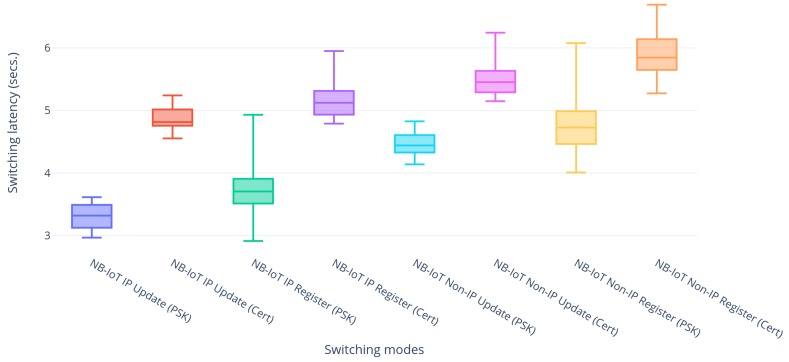
Handover latencies of NB-IoT with DTLS using Pre-Shared Key (PSK) and certificates.

After having introduced some typical latencies for LwM2M registrations and updates over NB-IoT and BLE individually, this subsection further continues to assess this behavior when performing actual handovers. All combinations regarding modes have been explored and the results are shown in Figure 5, Figure 6 and Figure 7.

Figure 5 shows the handover latencies of NB-IoT and BLE for all the possible modes. It can be observed that in general the latencies of NB-IoT are higher than those of BLE. This is due to the fact that NB-IoT works in the sub-GHz spectrum at lower data rates, resulting in higher transmission times compared to BLE which works in the 2.4 GHz spectrum. Ideally, BLE IP is simply 6LoWPAN on top of BLE connectivity, therefore expecting almost similar performances as those of BLE Non-IP. This is not the case, due to performance issues with the BlueZ stack [23] on which the BLE IP implementation is based. Therefore, BLE Non-IP has been taken as a frame of reference for a fair comparison. Another difference that is noted is the one between the latencies for registration and update messages. Default registration messages of our LwM2M client require 149 bytes for uplink and 26 bytes for downlink, whereas update messages have 26 bytes for uplink and 12 bytes for downlink resulting in the registration messages experiencing higher latencies than update messages. The deviations for the latencies in NB-IoT are higher due to the fact that the user equipment (UE) can be either in RRC connected or RRC idle mode at the instant of transmission. Therefore, the UE has to get into RRC connected mode before the actual transfer of data. Moreover, for each transmission, the UE has to get a grant from the eNodeB which leads to a scheduling delay. Other network dependent factors like signal strength, Modulation and Coding Scheme (MCS), Transport Block Size (TBS), scheduling algorithm used, etc. which are not under control of the UE add to the variations of the latencies. BLE on the other hand makes use of a fixed connection interval, configured to be 7.5 ms in our experiment, leading to more deterministic latencies and less deviations. NB-IoT non-IP has slightly greater latencies than NB-IoT IP due to the fact that the non-IP path needs to pass via the VNO, which performs some processing on the packets, thus adding to the latency in either direction. The same behavior could not be observed for BLE due to the limitations of the BlueZ stack as mentioned before. Lastly, SCHC adds to the latency by almost 100 ms due to the compression and decompression computation latencies in either direction in our set-up. It runs on top of the non-IP implementation and hence we see the constant latency difference of SCHC with respect to non-IP. So in general it can be summarized that BLE non-IP has the lowest latency followed by BLE SCHC, BLE IP, NB-IoT IP, NB-IoT non-IP, and finally NB-IoT SCHC.

DTLS is a security mechanism typically applied on IoT use cases that make use of the UDP transport protocol for their data exchange. Therefore, its effect on the handover latencies should be taken into consideration. DTLS comes in various flavors out of which the PSK and certificate modes have been applied on the set-up. Figure 6 shows the comparative latencies of handover to BLE with and without using DTLS in PSK mode. In general it is observed that the latencies introduced by DTLS are much higher compared to the case without security. This is due to the fact that DTLS involves a handshake between the communicating entities for the authentication and key exchange before the actual transfer of the encrypted data. For PSK, the handshake requires five uplink messages and three downlink messages and the last pair of uplink and downlink message denotes the actual encrypted data transfer resulting in 10 messages in total for uplink and downlink. The overhead of the eight extra messages for the handshake results in the increased latency. The same phenomenon is observed for NB-IoT.

Lastly, the differences of applying DTLS in PSK and certificate mode have been studied, because the certificate mode provides more flexibility in terms of key generation in the event of a key compromise. Figure 7 shows the comparative latencies of using DTLS in the two modes for NB-IoT and shows that the certificate mode has greater latencies as compared to the PSK mode. This is due to the fact that the certificate mode has two extra message transfers for the transfer of certificates and the packet sizes are larger compared to PSK, resulting in 12 message transfers between the LwM2M client and server. Moreover, as the used Sara N210 NB-IoT module [8] has a limited application payload of 512 bytes, packets larger than that had to be fragmented in the application layer resulting in even more packets for the handshake establishment. The same also applies to BLE, but there the implementation has already layer 2 support for packet fragmentation, not requiring any application level fragmentation.

## 6. Analysis

The novelty of the multimodal approach lies in the fact that it puts together the advantages of NB-IoT and BLE. NB-IoT is targeted for long range with low data rates, whereas BLE is for short range with higher data rates. Therefore, essentially, BLE should be configured with the minimal connection interval of 7.5 ms as configured in our setup. This makes BLE ready for high data rates and low latencies with high availability. Thus, just maintaining the BLE connection consumes 0.45 C on average over a time frame of 59 s. This is almost equal to the RRC idle state of NB-IoT and should be considered as a drain of energy. However, as noticed, BLE has very short connection times, ~1.2 s, consuming a total of 9 mC of electric charge. Thus, for applications that are infrequent and delay tolerant for 1 s, one can switch off the BLE radio to save power and switch it back on whenever necessary, after which the data transfer can happen at a rapid speed with high energy efficiency. Use cases where the radio is normally silent, but where sudden burst of data at high data rates are required (e.g., firmware upgrade, emergency voice streaming, etc.) are of this category.

On the other hand, NB-IoT shows very stable performance with respect to varying signal strengths and the ability to deliver data with no packet losses, but at the expense of larger latencies and reduced data rate. The energy efficiency for a data transfer over NB-IoT is lower than the one of BLE. Moreover, NB-IoT incurs overheads for signaling, change of transmission and reception power based on channel conditions, repetitions, high connection times of 22.5 s and greater power consumption. Thus, NB-IoT should be used only in those cases where there is no BLE coverage or for delay tolerant applications. However, enabling PSM consumes very little energy and the device can still transmit anytime it wants. Therefore, depending on the use case the multimodal architecture can be configured accordingly, but most use cases will perform well with NB-IoT in PSM mode and BLE switched off and switched back on when necessary, provided the application use case is device initiated. For use cases that require constant downlink supervision, either NB-IoT or BLE should be kept active with properly configured connection intervals, slave latencies for BLE or eDRX and PSM for NB-IoT to conserve energy.

The results and conclusions drawn in this work complement existing research that consider the short range technology BLE and the long range technology NB-IoT separately. The state-of-the-art considers the performance evaluation of BLE and NB-IoT individually in terms of throughput, latency, and power consumption and shows the progress from one generation to the other. Moreover, it shows that different configuration parameters have a strong effect on the performance of a particular technology. However, it does not consider the possibilities of combining both to support more dynamic IoT applications, nor the impact of switching from one technology to the other. Further, our architectural design along with its implementation testifies that future IoT applications can indeed benefit from this type of flexible, easy-to-use and intelligent protocol stacks without the need to address heterogeneity and while still attaining seamless inter-operability.

## 7. Real Life Scenario

Bringing everything together, this section applies all findings to a typical scenario where handover between NB-IoT and BLE is performed. It is considered that an end device has the availability of both technologies NB-IoT and BLE, but that at a certain instant of time, the service or coverage of a certain technology may or may not be available. This can lead to two situations, the first one being that one technology becomes unavailable and the device needs to switch to the other one, further called a passive handover. The second one, is the one where both technologies are available and the device actively initiates a handover to make use of the best suited technology. These two cases are further discussed below.

### 7.1. Active Handover

**Figure 8:**
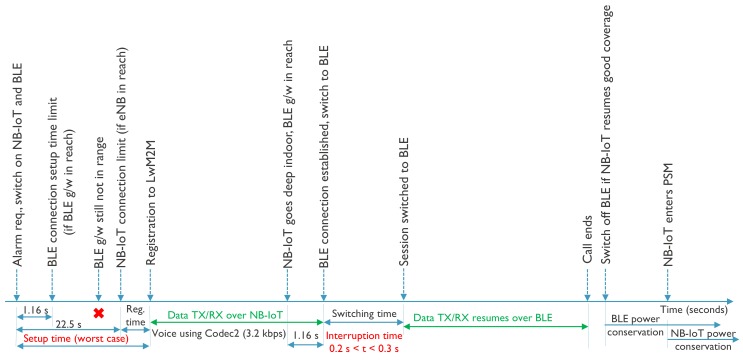
Active handover from NB-IoT to BLE.

As mentioned, an active handover is the process where the device itself takes the initiative to switch to another technology instead of continuing to use the technology currently being used. This can be due to the fact that the device notices a constant degradation of the signal strength for the current technology and after a certain threshold decides to seek for another technology before the service gets interrupted. Figure 8 shows a scenario where a device actively switches from NB-IoT to BLE. It demonstrates an emergency health-care use case, where a patient while being outdoor requires emergency medical support. Considering that both the NB-IoT and BLE radios are turned off, the emergency alarm triggers a connection initiation to both of them. As the patient is outdoor, chances of being in reach of a BLE gateway are feeble. NB-IoT can communicate over long distances, and therefore it becomes the active technology; however, it takes longer (~22.5 s) than BLE (~1.2 s) to attach to the nearest eNodeB. The data transfer then happens over NB-IoT as the patient is being taken to the hospital. Once the patient reaches the hospital, the signal strength of NB-IoT can become weaker because of deep indoor environments, but at the same time there is a higher probability of getting into the reach of a BLE gateway in the hospital. The device noticing the weaker NB-IoT signal strength and detecting the BLE signal, decides to perform an active handover to BLE and gets connected to a BLE gateway. Until now, the data transfer was uninterrupted, but as the device sends an update message to the LwM2M server over BLE and until it receives a response back, the data transfer is interrupted for around 300 ms after which the data transfer resumes over BLE. After the data transfer ends and assuming future necessities of emergency communication whilst being power efficient and availability of the NB-IoT signal, the BLE radio can be switched of and the NB-IoT radio can be put in PSM mode. This way, an emergency situation can be handled combining both availability and energy efficiency.

### 7.2. Passive Handover

**Figure 9:**
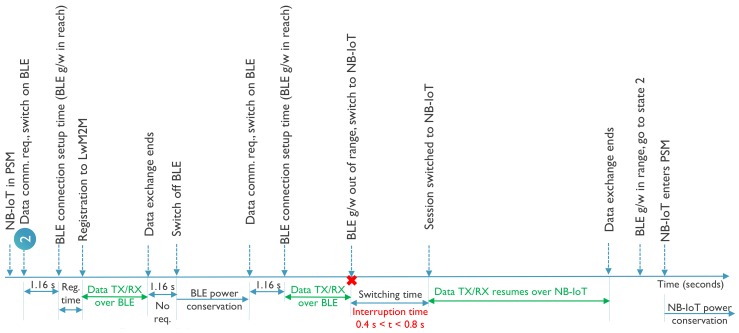
Passive handover from BLE to NB-IoT.

A passive handover is the case when the device using a particular technology continues to use it as long as it is available and only initiates a handover once the service is disrupted. Figure 9 shows the case of a passive handover from BLE to NB-IoT, including timings and time limits that have been observed during our experiments. It demonstrates the case of a scenario where an application generates burst of data at certain time intervals. As BLE has higher throughput and energy efficiency it is the preferred technology. Assuming a BLE gateway is within reach, the connection is established and data transfer takes place. To save power the BLE radio is switched off after the data transfer assuming that the BLE connection time is not critical for the application. However, at a certain instant during the data transfer the device moves outdoor and recognizes that the BLE gateway is no longer within range. It then initiates a handover to NB-IoT, connects to the eNodeB and the data transfer resumes. Assuming that NB-IoT was in PSM mode the handover time, which includes the registration update to the LwM2M server, can take up to 800 ms under good channel conditions. Once the device is within reach of the BLE gateway again, it can go back to BLE connectivity and let NB-IoT go into PSM mode. This way it can serve the purpose of the application with higher throughput and better energy efficiency but still having fail-safe mechanisms at times of need.

## 8. Conclusions and Future Work

In the wireless communication domain there are different technologies, each one having its distinguished characteristics regarding range, data rate, latency, availability, power efficiency, cost, etc. As such, the technology for a particular IoT application has to be chosen wisely. However, for some applications that change their requirements with time, that need a fail-safe mechanism or that want to optimize power consumption, there is the need for multiple technologies to be in place, giving rise to the notion of multimodal IoT. Multimodal IoT architectures gather the benefits of several technologies in one place and try to make optimal use of them, switching from one to the other as per the need. This leads to a flexible platform for devices to choose from the technologies and opens up a wide number of useful applications in the IoT world. In this paper two technologies, namely, NB-IoT and BLE, have been integrated into such a multimodal architecture. The purpose of choosing these two technologies is that they serve extremes, with NB-IoT having long range and deep indoor coverage while BLE having higher data rates and a high energy efficiency. As such, the multimodal model has the capability to serve dynamic IoT applications in an optimal fashion. The model has been designed and prototyped with an end-to-end working platform and evaluated in terms of the overhead incurred due to the handover in terms of the latency, thereby assuming LwM2M/CoAP as the common higher layer application protocol. This gives rise to the possibility of using either of them, and to choose the appropriate one based on the priority of the parameters among which response time and energy consumption play the most important roles. The insights from our study lead to the possibility of designing and applying efficient multi objective optimization (MOO) algorithms for multimodal architectures with the possible usage of cognitive infocommunications for smarter and smoother functioning of dynamic context-aware IoT applications.

## Figures and Tables

**Table 1:** Table for data sizes and direction for different LwM2M events.

LwM2M Event	Description	Uplink Data Size (Bytes)	Downlink Data Size (Bytes)
**Reg_nosec**	Registration with no security	149	26
**Update_nosec**	Update with no security	26	12
**Reg_PSK**	Registration using PSK	255 + 287 + 164 + 14 + 93 + 221	60 + 292 + 107 + 93
**Update_PSK**	Update using PSK	255 + 287 + 164 + 14 + 93 + 93	60 + 292 + 107 + 77
**Reg_cert**	Registration using certificates	419 + 451 + 628 + 159 + 99 + 14 + 93 + 221	60 + 822 + 107 + 93
**Update_cert**	Update using certificates	419 + 451 + 628 + 159 + 99 + 14 + 93 + 93	60 + 822 + 107 + 77

**Table 2:** Table for NB-IoT latency for different LwM2M events.

NB-IoT IP LwM2M Event	Expected Latency * (s)	Measured Avg. Latency (s)	Error %
**Reg_nosec**	0.4015	0.55	−26.9981
**Update_nosec**	0.3162	0.5	−36.7571
**Reg_PSK**	1.8731	3.7	−49.3762
**Update_PSK**	1.7837	3.6	−50.4541
**Reg_cert**	3.1282	5.2	−39.8415
**Update_cert**	3.0388	5	−39.2238

* Calculated with No. of uplink repetition = 1 and downlink repetition = 4 at RSSI −75 dBm with MCS 7($I_{TBS}$).

**Table 3:** Table for BLE latencies for different LwM2M events.

BLE Non-IP Event	Expected Latency * (s)	Measured Avg. Latency (s)	Error %
**Reg_nosec**	0.2366	0.27	−12.3672
**Update_nosec**	0.2365	0.25	−5.3993
**Reg_PSK**	1.92	1.8	6.6667
**Update_PSK**	1.92	1.75	9.7143
**Reg_cert**	1.9725	2.15	−8.2558
**Update_cert**	1.9725	2.05	−3.7805

* Assuming 30 connection intervals between uplink and downlink transfers.

## Abbreviations

The following abbreviations are used in this manuscript:

LwM2M	Light-weight Machine to Machine
NB-IoT	Narrow-band Internet of Things
BLE	Bluetooth Low Energy
SCHC	Static Context Header Compression
